# From “Animal Machines” to “Happy Meat”? Foucault's Ideas of Disciplinary and Pastoral Power Applied to ‘Animal-Centred’ Welfare Discourse

**DOI:** 10.3390/ani1010083

**Published:** 2011-01-11

**Authors:** Matthew Cole

**Affiliations:** Faculty of Social Sciences, Open University, UK; E-Mails: m.d.d.c.cole@open.ac.uk; matthew.cole@vegatopia.org

**Keywords:** ‘animal-centred’ welfare, disciplinary power, Foucault, ‘happy meat’, pastoral power, Welfare Quality®

## Abstract

**Simple Summary:**

This paper considers some recent developments in ‘animal-centred’ welfare science, which acknowledges the sentience of ‘farmed’ animals, alongside the emergence of a market for ‘happy meat’, which offers assurances of care and consideration for ‘farmed’ animals to concerned consumers. Both appear to challenge the instrumental ‘machine’ model characteristic of ‘factory farming’. However, in both cases, this paper argues that these discourses of consideration for the well-being of ‘farmed’ animals work to appease and deflect ethical concerns while facilitating the continued exploitation of ‘farmed’ animals.

**Abstract:**

Michel Foucault's work traces shifting techniques in the governance of humans, from the production of ‘docile bodies’ subjected to the knowledge formations of the human sciences (disciplinary power), to the facilitation of self-governing agents directed towards specified forms of self-knowledge by quasi-therapeutic authorities (pastoral power). While mindful of the important differences between the governance of human subjects and the oppression of nonhuman animals, exemplified in nonhuman animals' legal status as property, this paper explores parallel shifts from disciplinary to pastoral regimes of human-‘farmed’ animal relations. Recent innovations in ‘animal-centred’ welfare science represent a trend away from the ‘disciplinary’ techniques of confinement and torture associated with ‘factory farms’ and towards quasi-therapeutic ways of claiming to know ‘farmed’ animals, in which the animals themselves are co-opted into the processes by which knowledge about them is generated. The new pastoral turn in ‘animal-centred’ welfare finds popular expression in ‘happy meat’ discourses that invite ‘consumers’ to adopt a position of vicarious carer for the ‘farmed’ animals who they eat. The paper concludes that while ‘animal-centred’ welfare reform and ‘happy meat’ discourses promise a possibility of a *somewhat* less degraded life for *some* ‘farmed’ animals, they do so by perpetuating exploitation and oppression and entrenching speciesist privilege by making it less vulnerable to critical scrutiny.

## 1. Introduction

In this paper I use Foucault's ideas of disciplinary and pastoral power [1,2] to try to understand a discursive shift in the way that ‘farmed’ animals [3] are understood in Western culture. In 1964, Ruth Harrison introduced the phrase ‘animal machines’ in a book of the same name to describe how ‘factory farming’ had reduced its nonhuman victims to the status of units of productivity [8]. This ‘mechanisation’, or objectification, of nonhuman animals depended on a Cartesian denial of their sentience, emotion and communicative capacity: “a cultural logic that defines them as ‘dumb,’ as lacking the ability to express themselves, or indeed lacking any self to express” [9]. Since that time, and not least due to the impact of *Animal Machines* itself [10], public disquiet has grown about the way of relating to and conceiving of, ‘farmed’ animals that ‘factory farming’ implies.

One response to the troubling notion of animal machines is that of ‘happy meat’—the belief that it is possible to raise and kill animals in such a way as to remove the ethical problems associated with the ‘machine’ discourse of ‘factory farming’—to hear the expression of the animal Other, yet continue to confine and kill. This belief in the possibility of ‘happy meat’ has its academic counterpart in ‘animal-centred’ welfare discourses, in which an argument for the intelligibility of nonhuman animal well-being through observation of their expressive behaviour is advanced [11]. This is not to argue that ‘happy meat’ is replacing ‘animal machine’ discourse in practice—the vast majority of ‘farmed’ nonhumans still endure their short lives in ‘intensive’ systems. However, the emergent ‘animal friendly’ discourses, both in popular and academic culture, does suggest a change in the way that humans conceive of their relationship with ‘farmed’ nonhumans. Welfare reforms motivated by a belief in nonhuman animal sentience and their capacity to experience and express a complex emotional life *could* ameliorate suffering for some ‘farmed’ animals. However, the well-being of ‘farmed’ animals is incidental to this discursive reconfiguration of relationships between humans and ‘farmed’ animals. In conceding sentience and an expressive self, while *continuing to confine and kill* for gustatory pleasure, ‘happy meat’ and ‘animal friendly’ welfare discourses attempt to remoralize the exploitation of ‘farmed’ animals in such a way as to permit business as usual, with the added ‘value’ of ethical self-satisfaction for the consumer of ‘happy meat’. This discursive shift then, is concerned with reconfiguring the self-concept of human consumers of nonhumans, so as to repel the challenge that a shaken faith in the mechanistic view of ‘farmed’ animals poses and to restore a sense of rightful, but ‘benevolent’, domination of nonhuman animals. ‘Happy meat’ is very bad news for the goal of animal liberation [12].

In the next section of this paper I briefly consider the general applicability of Foucault's work to human-nonhuman animal relations, before exploring in more detail some of the resonances and tensions between Foucault's work on disciplinary power and the ‘factory farming’ model. I then turn to academic ‘welfare friendly’ discourses and practices and consider them in light of Foucault's account of pastoral power, before considering the popular cultural expression of ‘welfare friendly’ academic discourse: ‘happy meat’. This is followed by a consideration of the reconfiguring of the ‘human’ in relation to the shifting ‘Other’ that is implied in the move from disciplinary to pastoral accounts of human-‘farmed’ animal relations. I conclude with some reflections on how liberation activism and scholarship might respond to this new discursive challenge.

## 2. Foucault and Nonhuman Animals

Foucault's theorizations of disciplinary and pastoral power have been used to trace shifting tendencies in the governance of humans, from the production of ‘docile bodies’ subjected to the knowledge formations of the human sciences (disciplinary power—see, for instance, Foucault's own *Discipline and Punish* [2]) to the facilitation of self-governing agents directed towards specified forms of self-knowledge by quasi-therapeutic authorities (pastoral power [14,15]).

Foucault's own work evinces little interest in nonhuman animals however, with the partial exception of sections of *Madness and Civilization* [16], wherein, ‘Foucault's discourse of animality is … largely symbolic and imaginative, and has little or no contact with animals understood as living biological organisms’ [17]. The human-nonhuman power relationship inherent in pastoralism is also only discussed by Foucault as a metaphor for human-human relations [1]. Furthermore, there are important differences between the governance of human subjects and the oppression of nonhuman animals. Most obviously, the latter are generally denied possession of themselves (exemplified in their legal status as property, see Francione [18]), or denied equivalent moral worth to human animals even when a level of subjectivity is granted to them, as in the case of their continued instrumentalization in animal welfare science (see Twine [19]).

Nevertheless, lack of overt interest in human-nonhuman animal relations by a theorist, does not disallow the development of that theory by others in that direction. For instance, ‘classical’ Marxist concepts have recently been developed to analyse the human exploitation and oppression of nonhuman animals by David Nibert [7] and Bob Torres [20]. Meanwhile, scholars and activists such as Carol J. Adams [21] and Marti Kheel [22] have applied the tools of feminist theory to understand the intersections of gendered and specied oppression to influential effect. Palmer argues that ‘Foucault's … work on the repressive and creative effects of power on bodies offers a range of tools for thinking through … human/animal relations’ [17]. Recently, those tools have been taken up in respect of ‘farmed’ animals [19,23,24,25,26,27,28]. In the next section I take Palmer's cue and discuss the relevance of ‘disciplinary power’ to understanding the lives of ‘animal machines’, firstly through consideration of some of the practices of ‘factory farming’ and secondly in the context of contemporary discourses of welfare in ‘animal science’ journals.

## 3. Disciplining ‘Animal Machines’

There are a number of features of Foucault's account of disciplinary power that appear to ‘fit’ with the experience of nonhumans in industrialized ‘farming’ systems. A key feature of disciplinary power is what Novek terms the ‘efficient regimentation of docile bodies’ [27] (see also Coppin, [23]), especially through material structures, technologies and the imposition of bureaucratic knowledge gathering procedures. Notable institutional sites for the emergence of disciplinary power in nineteenth century Western society included prisons, hospitals, schools and barracks, wherein inmates were variously subject to correction, cure, education and drill in relation to norms of lawfulness, health, employability and obedience to military authority [2].

Novek argues that intensive ‘farms’ are comparable to these sites in respect of their production of ‘docile’ bodies [27]. He gives the example of intensive ‘hog production’ in Canada, wherein pig's bodies are disciplined through confinement technologies (indoor ‘housing’, sow stalls, gestation stalls and so on) in relation to norms of maximum ‘productivity’ (the fastest possible growth of saleable body tissues) and ‘reproductivity’ (the fastest possible breeding cycle). Erika Cudworth notes that ‘docility’ is also an explicitly sought after and marketed characteristic of ‘farmed’, especially female, animals, in the discourses of breeders, especially as it implies ease of handling (*i.e.*, control) for their human captors [29]. Docility, argued Foucault, depends on ‘the distribution of individuals in space’ [2] such that they are amenable to surveillance and the production of individualised knowledge. The cellular arrangement of animals in cages, crates and stalls achieves that end, facilitating the monitoring and correction of poor ‘performance’ (and ‘correction’ may ultimately entail death if profits are compromised by the cost of veterinary care).

Many intensive ‘farming’ practices are similarly suggestive of the production of docile bodies through spatial distribution, surveillance and ‘correction’. Technologies of confinement such as battery cages, ‘broiler’ sheds or ‘veal crates’ all share the motif of ‘correcting’ nonhumans for their ‘wasteful’ use of energy (read: feed, for which read: economic cost) to sustain their own biological processes. Mutilations are designed to ameliorate the economic costs of ‘aggression’ that result from confinement technologies (that is, result from the actions of the human captors who design, build and maintain them), such as the amputation of horns, tails or beaks or the ‘clipping’ of teeth. Other mutilations discipline the flesh of nonhumans in relation to norms of human taste preference, for instance the castration of male piglets to prevent unprofitable ‘boar taint’. Sexual behaviour and the reproductive process is controlled through forcible artificial insemination (more properly described as rape [21,29]). Identification through technologies such as branding or ear-tagging make animals' bodies available to individualised surveillance and knowledge gathering (for further description of many of these techniques, see Marcus [30]).

Other disciplinary technologies have the appearance of benevolence, or at least mercy, compared to the acts of imprisonment and torture described above, but are still oriented towards inculcating docility: the use of stunning techniques in slaughterhouses facilitates an increase in the pace of dismemberment and therefore an increase in profitability; innovations to make slaughterhouses less ‘stressful’ environments for nonhumans similarly facilitate more saleable body parts due to decreased levels of adrenalin and ‘tastier’ flesh (see Smith for a critique of ‘humane’ slaughter [31]). As Foucault argued, disciplinary power individualises at the same time as it homogenizes: ‘[i]t allows both the characterization of the individual as individual and the ordering of a given multiplicity’ [2]. Intensive ‘farms’ facilitate the surveillance and traceability of particular animals; a body of knowledge can be built up about their ‘performance’ in relation to norms of productivity, reproductivity or the absence of disease. But at the same time, each animal is an interchangeable representative of a particular species, breed or strain of ‘food animal’ with a particular ‘function’ (breeding, fattening, lactating, egg-laying, *etc.*). The records of individual performance in turn contribute to the normalisation of the animal-as-multiplicity, for instance through selective breeding in light of the accumulation of knowledge about individual animals.

This combination of normalisation on the level of individual and ‘population’ is exemplified in the experiments of scientists working in the field of ‘animal welfare’. Beyond refining the technologies of restraint, incapacitation and killing that have been integral to the development of factory ‘farms’, animal welfare science also works on the *personality* of ‘farmed’ animals so as to render them more docile: Ghareeb *et al.* [32] describe the TI (Tonic Immobility) test as an assessment tool for the ‘individual personality characteristics’ of hens. The test involves hens being manually held down on the ground of a testing arena (that is, on their backs under the pressure of a human hand on their breast) and timed as to how long they remain immobile after the restraint has been removed. The longer they remain immobile, the more fearful they are interpreted as being. Fearfulness therefore translates into docility: “a highly fearful individual may be more social with its [*sic*] companions” [32]. The goal of such *individualised* tests is to inform selective breeding and reconstitute *whole flocks* of hens, to “help farmers or breeders to select birds with more desirable personality traits” [32]. The technique of modifying the ‘personality’ of ‘farmed’ animals therefore represents an attempted reconciliation between the material structures of confinement and the bodies of the confined, always in relation to the norm of ‘productivity’:

…there should be genetic selection for behavioural traits like sociality relevant for adaptation to particular housing systems, because a mismatch between underlying sociality and the bird's social environment elicits either acute stress responses or chronic distress, which damage welfare and productivity (emphasis added) [32].

The experiments of Ghareeb *et al.* are not an isolated example. As Turner *et al.* write, “[t]here is increasing interest in genetic selection against behavioural traits that impact negatively on welfare *and productivity* in commercial livestock production” (emphasis added) [33]. The significance of an emotional experience for ‘farmed’ animals in welfare discourse is always tempered by the situating of that experience within the logic of productivity. While breeding fearful hens is justified as being for their own good, fear is simultaneously treated as undesirable if it impacts on ‘meat quality’:

[A] relationship was demonstrated between animals' relationship to humans and their performance and product quality. For instance, studies in calves and lambs show possible positive effects of less fear of humans and handling on glycolytic potential, ultimate meat pH, and meat quality [34].

Animals' relationship to humans, *i.e.*, their perception of humans, considerably affects animal welfare and production […] Cattle being more fearful of humans, for instance, show acute and chronic stress responses, lower milk yield and reduced milk let-down [35].

Pigs reared in a barren environment showed more distress during fattening […] had a greater cortisol response during transport […] and had poorer meat quality compared with pigs reared in an enriched environment [36].

The juxtaposition of ‘welfare’ and both quantitative and qualitative measures of ‘productivity’ in these examples, all taken from recent animal welfare journals, is striking and a further point of fit with disciplinary power. The human disciplines are also legitimated with a discourse of welfare—to be reformed, cured, educated or well-drilled is argued to be for the benefit of the disciplined. Even the most brutal conditions of factory ‘farming’ are defended with the same ‘win-win’ discourse [28], notoriously in the figure of a captor (‘farmer’) in *The Animals Film* (1982) who, apparently in all sincerity, defends veal crates on the basis that they protect calves from predation (as if humans were not their primary predator) and extremes of weather. This kind of implicit invocation of a social contract between ‘farmer’ and ‘farmed’ animal is critiqued by Clare Palmer, who points out the inequality between the two parties, the irrevocability of any such contract from the nonhuman point of view, and the questionable nature of any advantage gained by the nonhuman party, especially given the inevitability of their early death at human hands [37]. The ‘protection’ discourse of ‘farmed’ animals is often deployed when animals are held captive far from the habitats to which they are adapted. As Novek points out, the intensive ‘farming’ of pigs in Canada is ‘excused’ because ‘[y]oung pigs, kept outdoors or in primitive shelters, simply could not survive Canadian winters’ [27]. Coppin argues that intensive pig ‘farming’ in ‘mega-hog farms’ in the USA is related to consumer demand for leaner meat, which necessitates pigs with lower body fat content. Pigs selectively bred for this characteristic therefore become dependent on ‘climate-controlled buildings’ for survival [23]. In ways such as these, ‘farmed’ animals are forced into dependency on human ‘care’ [17], an important technique in legitimating disciplinary power.

Despite these congruencies, a note of caution is needed in applying disciplinary power to the case of ‘factory farming’. Power always has a *relational, mobile and active* quality in Foucault's work. If the prisoner, patient, pupil or soldier did not have the capacity to resist their subjectification in relation to disciplinary norms, there would be no *relation* to speak of, no contestation of the process of normalisation: “[a] man who is chained up and beaten is subject to force being exerted over him, not power” [1]. The obverse of power, for Foucault, is therefore not freedom, but domination: “…an underside that is in the end always passive, doomed to perpetual defeat” [38]. A violent death is almost inevitable for ‘farmed’ animals, excepting rare escapes and rescues. Therefore, in situations where no resistance to that doom is possible, power relations appear problematic as a framework for understanding (for a similar cautionary note, see Twine [19]).

Coppin addresses this issue by arguing that if “we can see resistance at a biological level, then this opens the concept to a notion of nonhuman resistance” [23]. As an example of this biological resistance, Coppin goes on to describe pigs biting through wooden pen floors [23]. Similarly, Novek suggests that the very behaviours that are subject to the interventions of animal welfare science, such as stereotypies or tail-biting, are evidence of resistance to the norms of ‘factory farm’ discipline, ‘[p]igs, perhaps, have not been socialized fully to accept the disciplinary techniques that have been imposed on them’ [27]. Disciplinary power does not therefore provide an *alternative* to concepts such as exploitation or oppression in accurately describing human relations with ‘farmed’ animals, but a *complementary* set of tools for understanding the techniques of exploitation and oppression. While the dystopia of the perfectly docile meat-machine remains the telos of ‘factory farm’ discipline (the achievement of which would constitute a state of domination in Foucault's terms), the very existence of the panoply of disciplinary techniques demonstrates that perfect docility is a long way from being achieved. Richard Twine suggests that the domination telos is elusive as a consequence of the biological limits of ‘farmed’ animals, circumscribing their exploitability, as manifested in the prevalence of mastitis or lameness among ‘dairy cows’, or weak bones among egg-laying hens for instance [28]. The lives of ‘farmed’ animals then, are not (yet?) utterly dominated.

Despite the ‘for their own good’ arguments that are used to justify disciplinary apparatuses like farrowing crates, awareness of the distress of confined animals and the mutilations to which they are subject inhibits the possibility for conceiving of their situation as beneficent, and therefore keeps open an arena of ethical doubt for meat-eaters. If there were no such doubt, it would be difficult to understand why ‘factory farms’ and slaughterhouses are so difficult to access [39]. The manifest tension between treating other animals as if they were machines while making claims about their welfare as if they were feeling, suffering, beings, begs for resolution. As Smith puts it, “to hear their [‘farmed’ animals'] voice as a form of self-expression, as a language that might speak to us, that might alter our sensibilities, would be to jeopardise our special status, our separateness” [31].

How much more economical it would be if power relations could be configured in such a way as to give the impression that interventions in the lives of others were really in accordance with their natures, needs and wishes. Thus: pastoral power, ‘animal centred’ welfare reform and ‘happy meat’.

## 4. Pastoralism and ‘Animal Centred’ Welfare

Since the publication of Ruth Harrison's book Animal Machines in 1964, public opinion with regard to animal welfare has much changed. Animal welfare is considered a physical and mental state related to the absence of negative emotions […] Much effort has been made to reduce stress, and thus to improve animal welfare during the rearing period [10].

The emotional turn in public animal welfare discourse that Terluow *et al.* describe, has demanded a reconfiguration of human-‘farmed’ animal relations, a deprivileging of mechanistic discourses and a valorisation of the possibility of empathetic knowledge. Foucault's account of pastoral power provides a useful framework for understanding this discursive shift.

Before considering its application to ‘animal centred’ welfare discourse in detail, it is important to note that pastoral and disciplinary power are in a sense different aspects of a more general reconfiguration of power relations in the modern West, and in particular the rise of ‘biopower’, contingent on the demands of governing populations in the new context of urban industrial capitalism in the nineteenth century [38]. Rather than incompatible forms of governance, disciplinary and pastoral powers are perhaps better understood as heterogeneous assemblages of techniques for conducting life in modernity. The prominence of one or the other in a particular context depends on their efficacy in relation to the demands of the situation, not on epochal transformations in the form of governance. For example, the trend towards quasi-therapeutic discourses of unemployment in place of more overtly disciplinary stigmatizing discourses (the substitution of the ‘jobseeker’ for the ‘dole scrounger’) does not signal a grand narrative in the direction of greater societal benevolence, but a shift in the meaning of ‘work’ and therefore of the types of working subjects that are demanded [40]. Similarly, the growing concern with nonhuman animals' ‘feelings’ in animal welfare discourse [41] is not necessarily a chapter in a story that has animal liberation as its telos. Both disciplinary and pastoral techniques are simultaneously concerned with the governance of whole populations (biopolitics) and with individuals (anatamo-politics) [42], but, as I will illustrate below, pastoral power in ‘animal centred’ welfare evidences a shift in emphasis towards care for the subjective emotional states of ‘farmed’ animals.

As noted above, Foucault's discussion of pastoral power evidences species-blindness. He is interested in pastoralism only as a metaphor for human governance that emerges in the ancient cultures of the Middle East, especially in its early Christian manifestation [1] Foucault then goes on to trace the role of pastoral power in the formation of the modern state [1] (see also Golder [43]). In a sense then, the task of interpreting pastoral power in the context of human-‘farmed’ animal relations ought to be more straightforward. However, modern welfare discourse inhabits a very different world to ancient herding cultures [44]. Therefore, pastoralism differs from discipline in operating as a metaphorical description of power relations.

Foucault identified four characteristics of pastoral power, which shape the subjectivity of the figure of pastor as much as that of the ‘flock’ [1]. Firstly, *responsibility*, referring to the duty the shepherd has for ‘his’ flock as a whole, and for each individual sheep (thereby evidencing the bipolarity of biopower and a similar combination of individualization and homogenization to that noted above in the context of disciplinary power). Foucault stresses that the shepherd bears a moral responsibility not only for ‘individuals’ lives but the details of their actions as well' [1]. Secondly, *submission*, ‘the sheep must permanently submit to their pastors’ [1] and this submission should have the character of a self-willed obedience. Thirdly, *individualised knowledge*, which refers not only to the duty of the shepherd to know, but also the connection between ‘total obedience, knowledge of oneself, and confession to someone else’ on the part of each member of the ‘flock’ [1]. Fourthly, *self-mortification*: ‘[m]ortification is not death, of course, but it is a renunciation of this world and of oneself … a death that is supposed to provide life in another world’ [1]. Pastoralism therefore already offers the promise of solving the moral dilemma of disciplinary power relations that teeter between machine and welfare discourses of ‘farmed’ animals. The four characteristics of pastoral power together frame an intimate relationship between shepherd and flock in which *both* bear a moral responsibility for the subjection of the latter. How then does this play out in the context of ‘animal centred’ welfare discourse?

The Welfare Quality® (WQ) project is one manifestation of a trend towards quasi-therapeutic ways of claiming to know ‘farmed’ animals, in which the animals themselves are co-opted into the processes by which knowledge about them is generated [46]. WQ is a research project with a budget of 17 million Euros, substantially funded by the European Union, incorporating 44 partners in 13 European and 4 Latin American countries. A full description of the WQ project is beyond the scope of this paper (for an overview, see Blokhuis, [47]). The relevant point to the (re)emergence of pastoral power relations is its attempt to reconcile public and scientific discourses of ‘farmed’ animal welfare (and it is important to note that these discourses do not necessarily spontaneously coincide), through a combination of social research on ‘consumer’ attitudes and scientific innovations in ‘animal centred’ monitoring and assessment schemes on ‘farms’ and in slaughterhouses:

The standards for on-farm welfare assessment and information systems have been be [*sic*] based upon consumer demands, the marketing requirements of retailers and stringent scientific validation. The key was to link informed animal product consumption to animal husbandry practices on the farm. The project therefore adopted a “fork to farm” rather than the traditional “farm to fork” approach [48].

WQ therefore both responds to, and reinforces, the adoption of a quasi-pastoral role on the part of some ‘consumers’. It seeks to understand ‘consumer concerns’ and the ‘information demanded’ about ‘food animal welfare’ and enable concerned ‘consumers’ to act on that information through techniques such as labelling schemes that communicate welfare standards in the retail environment [47].

It is important to note that WQ's own research findings suggest that denial and lack of knowledge about the conditions of ‘farmed’ animals' lives and deaths is the norm among ‘consumers’ [49,50]. Such individuals are ‘eager to delegate responsibility for animal welfare to other actors, such as supermarkets or the state’ [49]. There is no reason to believe, therefore, that disciplinary power relations will come under serious pressure to be supplanted *en masse* as long as denial persists. Lack of awareness is frequently attributed to discomfort with the ethical problems that consuming other animals raises [49]. Some respondents however, demonstrated spontaneous interest in animal welfare issues, and when presented with ‘animal centred’ welfare discourses, ‘consumers’ frequently found the proposals appealing [49].

Limits of space prevent full discussion of WQ's proposals for welfare assessment and monitoring (more details can be found on the WQ website: http://www.welfarequality.net/everyone), but the following examples, taken from a WQ presentation and factsheet, are illustrative:

The welfare of an animal depends on how it [*sic*] experiences the situation in which it [*sic*] lives. The Welfare Quality® assessment scheme emphasises the animal's point of view by placing increased importance on measures taken on animals (e.g., bodily condition, injuries, fear) in its assessment [51].

Negative emotions such as fear, distress, frustration or apathy should be avoided whereas positive emotions such as security or contentment should be promoted [52].

Despite the persistence of the degendering use of ‘it’, these examples represent are striking in their claims that ‘positive emotions’ can be *promoted* by welfare regimes and furthermore, that the ‘farmed’ animals themselves *contribute* to improving their own conditions through the knowledge they can provide to WQ assessors. Recalling the four characteristics of pastoral power, WQ discourse here illustrates the beneficent form of *individualised knowledge* by which animals confess the truth of their experience as an integral aspect of their *submission* to their confessors.

The potential for ‘farmed’ animals to ‘tell’ about their subjective states is the leitmotif of the development of preference, or choice, tests in ‘animal centred’ welfare science (for overviews, see [41,53]). These have been developed as a way to infer the ‘feelings’ of ‘farmed’ animals, or what those animals do or do not want [53]: ‘The animal is allowed to choose between certain aspects of its [*sic*] environment, and the assumption is that the animal will choose according to how it [*sic*] feels’ [41]. In this way, ‘farmed’ animals are placed in a confessional relationship by which *individualised knowledge* is generated: ‘[b]ehaviour is […] the result of all of the animal's own decision-making processes’ [53]; ‘[p]reference testing is only the first step in ‘asking’ an animal what it feels’ [41]. Tellingly, Duncan celebrates research that found that ‘male broilers [*sic*] ate more of [… a] drugged feed than did broilers with no lameness, and ate enough of it to improve their lameness’ [41]. The birds therefore ‘tell’ of their suffering, but the ‘animal centred’ welfare resolution of this issue is the bird's *self-mortification* (‘choosing’ to drug themselves) rather than their liberation from the relationship that caused that suffering in the first place.

Another recent innovation in ‘animal centred’ welfare science, Qualitative Behaviour Assessment (QBA) incorporates an understanding of ‘farmed’ animals as bearers of ‘agency’ [54]. QBA is “a method based upon the integration by observers of perceived animal behaviour expression, using descriptors such as ‘calm’, ‘aggressive’, ‘sociable’ or ‘indifferent’” [55]. This is possible because QBA suggests that it is possible to interpret the expressions of other animals as a way to gain access to understanding the ‘whole animal’:

[T]aking the integrative nature of qualitative judgements seriously enables a ‘whole animal’ perspective, through which it becomes possible to view behaviour as a dynamic, expressive body language that provides a basis for assessing the quality of an animal's experience (e.g., contented, anxious). Judging this quality is a skill that requires knowledge of species-specific behaviour, experience in observing and interacting with animals in different contexts, and a willingness to *communicate with animals as sentient beings* (emphasis added) [11].

This passage describes how *individualised knowledge* can be generated within the context of a relationship defined by the *responsibility* of the human ‘judge’ of ‘farmed’ animal behaviour to empathize: “Such a perspective requires engagement with the animal's situation, and is essentially built on relationship and empathetic communication” [11]; “behaviour is […] a ‘body language’, which communicates *what it is like to be* that animal at a given moment in time” (emphasis in original) [11]. That relationship, and therefore the generation of *individualised knowledge*, also depends on the active input of those animals:

one of the ultimate objectives of animal-based monitoring … is to let the animals ‘say’/indicate what their actual level of enrichment is/has been, without having to rely on environment-based criteria and without having to rely on stockmen [*sic*] reports [56].

Pastoral power then, unlike disciplinary power, mobilises authoritative knowledge of the ‘whole animal’ in order to provide the certainty that ‘farmed’ animals really do enjoy good ‘welfare’, a certainty that is required if the pastor is able to fulfil her or his duties of *responsibility* and *individualised knowledge*. The discourse of QBA also evidences the pastoral subjection of ‘farmed’ animals through their own role in ‘confessing’ their whole being. *Self-mortification* and *submission* are less obviously integral to the broader discourses of WQ and ‘animal centred’ welfare, but become clear when placed in context. ‘Farmed’ animals do indeed practice *self-mortification* through an ‘everyday death’—the devotion of their lives to serving human appetites. None of their everyday activities make sense outside of the context of submitting to human desires for their flesh, skin, hair or secretions. Moreover, their eventual real deaths do, of course, provide life in ‘another world’: the human stomach. So, while innovations like WQ, choice tests or QBA offers the *possibility* of an improved quality of life for *some* ‘farmed’ animals, they do so from inside a tightly bounded discourse within which the legitimacy of seeking and claiming to ‘know’ ‘farmed’ nonhuman animals at all is unquestionable. It is only in the context of the instrumental use of ‘farmed’ animals that there is any imperative to ‘know’ them at all. Without human exploitation and oppression, the category ‘farmed animal’ would dissolve. Pastoral power then, is a reconfiguration of the *means* of exploitation that does nothing to challenge the *end* of exploitation itself. The truths that animal scientists are capable of interpreting from their obedient ‘flock’ are therefore strictly limited. There is a powerful vested interest in remaining insensitive to particular kinds of truth, for instance the ‘expression’ of the mere desire to *continue living and evade death*. The production of docility and removal of fear in the slaughterhouse, of course, makes it easier to remain deaf to the resisting voices of the condemned, which may be “best regarded as an extenuation of modernity's persistent failure to listen (attend) to animal Others” [9]. The normalisation of ‘farmed’ animals in relation to the display of ‘positive emotions’ or ‘appropriate behaviour’ further denies them the capacity to voice a complaint against the bare fact of being held captive and being treated as ‘resources’ and not ‘creatures’ [9].

What is especially interesting in WQ in particular is that its nascent fabrication of a pastoral nexus sucks in ‘consumers’ and ‘farmed’ animals, as well as other ‘stakeholders’ in exploitation and oppression: ‘farmers’, retailers, governmental regulators and so on [47]. In other words, ‘consumers’ also become subjected by pastoral power. Through making ‘welfare friendly’ purchases, and understanding the ‘knowledge’ about the ‘whole animal’ that may be communicated by a labelling scheme, consumers also assume responsibility for their, albeit deceased, ‘flock’. The great risk, from a liberationist or abolitionist perspective, is that WQ thereby offers a way for ‘consumers’ to ‘know’ about ‘farmed’ animal lives, to dispense with the ideological architecture of denial, to reconcile their ethical discomfort about meat-eating, and ultimately to continue consuming flesh at the same time as consuming the moral affirmation that they are fulfilling their duties in respect of ‘farmed’ animals. It is worth stressing that WQ explicitly does *not* position itself as in any way contributing to an incremental movement towards the eventual abolition of nonhuman animal use, by contrast, ‘Welfare Quality® will make significant contributions to the *societal sustainability* of European agriculture’ (emphasis added) [48]. Through their confessions of contentment, or at least absence of fear, the *submission* of ‘farmed’ animals is justified as being for their own good. The fundamentally exploitative nature of animal ‘farming’ is therefore made *discursively* less visible than it is in the *materially* hidden disciplinary regime of the ‘factory farm’. This is so insofar as the instrumental relationship with nonhuman animals remains unchanged, in which their bodies remain as exploited commodities, but the relationship is instead presented as beneficent and caring. In other words, the dilemma of ‘protect and eat’ [57] exemplified in the implicit ‘farmer’-animal contract critiqued by Palmer [37], is attenuated by pastoral power.

In the next section of this paper, I explore the impact that ‘animal centred’ welfare has on human relations with ‘farmed’ animals in the light of popular ‘happy meat’ discourse.

## 5. Caring for ‘Happy Meat’

As with scientific welfare discourses, whether ‘disciplinary’ or ‘pastoral’, popular ‘happy meat’ discourse always posits a win-win scenario: happy animals taste better, as these examples illustrate:

We are a specialist producer of superb free range, rare breed meat, where welfare is put first […] The result is quality tasty meat which you can trust. Our rare breed animals have a great life [58].

This isn't rocket science: an animal that has led a happy life produces great meat [59].

We know that an animal's quality of life impacts the quality of the meat we eat [60]; Relaxed livestock produce superior meat [61].

There is no reason you should be brutal to animals when you don't have to. It will bring bruises; it will bring adrenaline and bad hormones in the muscles (Ariane Daguin, of D'Artagnan, “one of the pioneers of humanely raised meats” [62]).

Consumers of ‘happy meat’ therefore receive a gustatory reward for their vicarious pastorship, as well as moral approbation for fulfilling their *responsibility* towards animals: ‘It can be expensive. And at farmer's markets, health-food stores, and restaurants everywhere, we're making the choice to spend a little more to eat—and feel—a lot better’ [62]. The very phrase ‘happy meat’ is curious when juxtaposed with ‘animal machines’. In the latter, a moral shock is produced from making explicit the process whereby subjects (animals) are transformed into objects (machines). The reader of Harrison's book is therefore invited to feel revulsion at the thought of consuming the results of this annihilation of subjectivity. By contrast, ‘happy meat’ imputes subjectivity (being ‘happy’) to an object (meat). This is literal nonsense, but useful to sustain the myth that pastoral power is not exploitative: the association of happiness with meat reinforces the idea that ‘farmed’ animals exist only to ‘provide’ meat. Being killed, butchered, sold and consumed is the fulfilment of their destiny, like the suicidal creature in Douglas Adams' novel *Restaurant at the End of the Universe*: ‘I am the main Dish of the Day. May I interest you in parts of my body?’ *Self-mortification* therefore becomes the reason for living, and dying, for these ‘pampered animals’ [62]. Popular food culture often deploys images of ‘suicidal’ or cannibalistic self-mortifying animals, often as cartoon characters smacking their lips at the thought of eating their own flesh, or that of one of their fellow creatures (for examples, see www.suicidefood.com). Happy meat makes this ‘vulgar’ process of representation ‘respectable’ through the architecture of pastoral power. In food writer Peter Rubin's extended article on the topic, ‘farmed’ animals already *are* meat: ‘[i]t’s [*sic*] animals *are* free-range, grass-fed, patiently raised; *artisanal meats*’ (emphasis added) [62]. It is not only ‘meat’ that can be happy.

One of the most prominent contemporary examples of the popularisation of pastoral power is UK-based Noble Foods, supplier of 60 million eggs per week to UK supermarkets [63] and owner of the ‘Happy Egg Company’ brand, which uses the ‘win-win’ slogan: ‘happy hens lay tasty eggs’. The Happy Egg website deploys anthropomorphized ‘quotations’ as captions for photographs of hens declaring how happy they are: ‘Bit of sunbathing with girls in the sandpit, perfect to sit back and relax with a cocktail or two!’ [64]. Needless to say, the fate of the ‘boys’, the slaughtered male chicks who are the ‘waste’ product of egg production, is absent from the website [65]. As with ‘happy meat’, happiness is a quality that is directly attributed to eggs themselves, not just the hens who lay them. A television advertisement states that, ‘happy eggs are wonderfully tasty’ and the branding deploys anthropomorphized cartoons of eggs with drawn on wings, beaks, eyes and so on [66].

‘Happy meat’ discourses therefore posit that happiness becomes an adjunct of meat (or for that matter eggs), something to be consumed along with the muscle fibres, fat and blood. The ‘ethical consumer’ is morally satiated by consuming the happiness of the animals at the same time as her or his belly is filled with their corpses or secretions. The juxtaposition of ‘welfare’ and ‘quality’ is therefore more significant than a legitimation of exploitation. Happy meat purveyors jump over themselves to promote both as selling points, sometimes to tragic-comic effect: On the same webpage, the Well Hung Meat Company (the virile discourse of meat-eating hardly needs comment here) boasts that, ‘Animal Welfare is top of our list of priorities’, but also claims that, ‘[m]ost importantly, taste is top of our agenda’. But the happiness doesn't stop there. Consumers of happy meat are also invited to assume a pastoral role *vis-à-vis* ‘farmers’ themselves, assuming caring responsibilities for the ‘little guy’ who works to preserve a noble (pastoral) way of life in defiance of the predominance of disciplinary agribusiness:

Exhaustive commercialization of the meat industry has resulted in innumerable, well-chronicled problems at large processors like ConAgra and Cargill: disease; cruelty; and insipid flavor that bears little resemblance to the poultry, beef, and pork of 50 years ago. But Veritas Farms and dozens of small producers around the country like it are presenting an alternative [62].

Happy meat also fetishizes ‘rare breeds’, imputing them with morally superior characteristics to the subjects of disciplinary power:

“They're great mothers,” Alward says. “They don't *need* to be crated in a farrowing crate, and chained in for six to eight weeks while they nurse so they don't roll onto their babies” (emphasis added) [62].

In this and other examples, claims for the aesthetically superior qualities of ‘happy meat’ animals (‘“Just look at him. Very princely” […] “It's like something out of Disney, right?”’ [62]) reflect credit on happy meat consumers, who claim vicarious responsibility for creating these beautiful, healthy and contented [*sic*] animals and dissociate themselves from the degradation of the ‘filthy animals’ of ‘factory farming’.

Breed fetishism leads Rubin to revisit the theological roots of pastoralism by writing of animals who are ‘resurrected from nearly extinct breeds’ and invoke metaphors of Noah when writing that ‘the farms can seem like rescue operations’. One of his interviewees goes further along a theological path in claiming that, ‘[i]f you believe there is a God, and you taste a very good meat, there is no way that this animal was made with that taste so that it could live without us tasting it’ [62]. This bizarre notion seems to suggest that we should all kill and eat as many animals as we can as quickly as possible in order to fulfil God's will. This is little different to the Marquis de Sade's justification for the rape, torture and murder of human and nonhuman victims in *The One Hundred & Twenty Days of Sodom* [67]. Sade posits a quasi-theology based on the idea of Nature as inherently destructive, the evidence being the manifest ubiquity of suffering and violence being inflicted by the strong on the weak in ‘nature’ [68]. The sadist's excuse is, therefore, that those who are privileged by a ‘natural’ social hierarchy have a duty to act in accordance with ‘nature’, and ‘destroy’ the unprivileged for their (sadists') own pleasure [67]. In other words, if it feels good, and we occupy a privileged position that lets us get away with it, do it! Ironically, the sadistic discourse of ‘nature as destructive’ presents a more honest assessment of the victimisation of the unprivileged than does the pastoral discourse of ‘care’ for the victims of ‘happy meat’ ‘farms’.

Happy meat discourse then, represents the ‘popular’ expression of pastoral power relations manifested in ‘animal centred’ welfare discourse. It facilitates adoption of the benevolent role of pastor in place of the disciplinary role of gaoler. It reassures consumers that they *know* the needs and desires of ‘farmed animals’, and that those needs and desires are being fulfilled precisely *because* they eat the flesh of those animals. The animals themselves therefore live self-mortifying lives of perfect submission to the aesthetic norms of their ‘consumers’.

## 6. Conclusions

In this paper I have outlined how the techniques of disciplinary and pastoral power present apposite frameworks for understanding a discursive shift towards ‘animal centred’ welfare and the emergence of ‘happy meat’. There is no explicit connection between the example of WQ and the ‘producers’ of ‘happy meat’ selected in this paper, but both evidence the emergence of pastoral power, respectively in a scientific and popular context. Importantly, WQ does indicate a political ambition to reconcile and mutually reinforce scientific and popular discourses of welfare, thereby excluding critical abolitionist and liberationist discourses from reshaping human-‘farmed’ animal relations in more radical and subversive directions. Deploying Foucault's work in this context delegitimates a welfarist narrative of moral progress as it highlights the *contingency* of ‘animal centred’ welfare: it is a form of pastoral power relationship *made necessary* by the growth of ethical concern with the role of human being as exploiter and dominator of the natural world, and other animals, partly in response to the efforts of advocates, activists and scholars in exposing the disciplinary horrors of ‘animal farming’. Disciplinary power relations, although they are what permeate the vast majority of ‘farmed’ animals' lives, are increasingly precarious and unsatisfactory for most people, as evidenced by WQ's ‘consumer’ research. However, ‘animal centred’ welfare raises more ethical problems than it solves (it solves none). A ‘farm’ or slaughterhouse achieving ‘success’ in an ‘animal centred’ welfare assessment indicates that ‘farmed’ animals have been *deceived* as to the murderous intentions of their ‘carers’. The ‘happier’ a ‘farmed’ animal the more profound is the betrayal of trust when the blood price of their ‘good treatment’ is extracted in the slaughterhouse. However, the process has the opposite effect on the ‘consumer’: the lack of discernible resistance, unlike in the cases of ‘aggression’ or stereotypies among factory ‘farmed’ animals, makes exploitation less visible and therefore less obviously vulnerable to ethical critique. As James LaVeck argues, ‘happy meat’ performs an Orwellian task of reconciling ethical qualms with continued desire for eating muscle tissue: ‘In a time not so long from now, practicing compassion will for many come to mean buying and eating happy meat, a purported win-win-win for the animals, the industry and its customers’ [69].

Veganism, which includes, but is not limited to, commitment to the renunciation of speciesist privilege, is the compassionate and rational response to the human oppression and exploitation of ‘farmed’ (and all other) animals. But it promises scant rewards for the privileged academic and economic interests that benefit from the continued subjugation of ‘farmed’ animals. To perpetuate that privilege requires new discourses, and pastoral power relations are so far proving effective towards that end:

Alward and Turco claim that a full 10 percent of their clientele are converted vegetarians and vegans who figure that eating clean meat does more to change the factory-farming industry than eating imported tofu [62] (see also www.humanemyth.org for testimonies and reports of vegan and vegetarian ‘converts’ to happy meat).

Writing critically on ‘happy meat’, Eddie Lama notes that:

For the meat industry, humane standards are good. Sales are up. It is also good for meat-eaters. They are made to believe they can now consume animals with a cleaner conscience. […]. But for me, it is a tragedy not only because more animals are being killed, but because a new culture of meat-eaters is being created. The concept of “Happy Meat” is entrenching a new system of animal exploitation into the world. It is making the killing of animals more acceptable to society's psyche and soul [70].

To remove that privilege therefore demands vigorous contestation of the architecture of pastoral power relations between humans and ‘farmed’ animals. The mere cultural visibility of ‘happy meat’ discourse may prove to be sufficient to appease the consciences of most meat-eaters that things are moving in the right direction, and that pastorship, with its attendant connotations of rural nostalgia for a ‘Golden Age’ of family ‘farms’, is the inevitable telos of contemporary welfare reform: ‘I've seen its life. I respect its death. And I feel okay’ [62].
